# Panel of three novel serum markers predicts liver stiffness and fibrosis stages in patients with chronic liver disease

**DOI:** 10.1371/journal.pone.0173506

**Published:** 2017-03-16

**Authors:** Marcin Krawczyk, Simone Zimmermann, Georg Hess, Robert Holz, Marc Dauer, Jochen Raedle, Frank Lammert, Frank Grünhage

**Affiliations:** 1 Department of Medicine II, Saarland University Medical Center, Saarland University, Homburg, Germany; 2 Laboratory of Metabolic Liver Diseases, Department of General, Transplantation and Liver Surgery, Medical University of Warsaw, Warsaw, Poland; 3 Roche Diagnostics, Grenzach-Wyhlen, Germany; 4 Department of Medicine III, Westpfalz-Klinikum, Kaiserslautern, Germany; 5 Department of Gastroenterology and Oncology, RKN-Hospital, Grevenbroich, Germany; University of Navarra School of Medicine and Center for Applied Medical Research (CIMA), SPAIN

## Abstract

Latest data suggest that placental growth factor (PLGF), growth differentiation factor-15 (GDF-15) and hepatic growth factor (HGF) are involved in hepatic fibrogenesis. Diagnostic performance of these markers for non-invasive liver fibrosis prediction was evaluated based on liver histology and stiffness. In total 834 patients were recruited. Receiver-operating-characteristics were used to define cut-offs for markers correlating to fibrosis stages. Odds-ratios were calculated for the presence/absence of fibrosis/cirrhosis and confirmed in the sub-group of patients phenotyped by elastography only. Logistic and uni- and multivariate regression analyses were used to test for association of markers with liver fibrosis stages and for independent prediction of liver histology and stiffness. Marker concentrations correlated significantly (P<0.001) with histology and stiffness. Cut-offs for liver fibrosis (≥F2) were PLGF = 20.20 pg/ml, GDF15 = 1582.76 pg/ml and HGF = 2598.00 pg/ml. Logistic regression confirmed an increase of ORs from 3.6 over 33.0 to 108.4 with incremental (1–3) markers positive for increased liver stiffness (≥12.8kPa; all P<0.05). Subgroup analysis revealed associations with advanced fibrosis for HCV (three markers positive: OR = 59.9, CI 23.4–153.4, P<0.001) and non-HCV patients (three markers positive: OR = 144, CI 59–3383, P<0.001). Overall, serum markers identified additional 50% of patients at risk for advanced fibrosis presenting with low elastography results. In conclusion, this novel combination of markers reflects the presence of significant liver fibrosis detected by elastography and histology and may also identify patients at risk presenting with low elastography values.

## Introduction

To date staging of liver status in patients with chronic liver diseases is mostly accomplished by liver biopsy [1–3]. Nevertheless, liver biopsy is associated with possible complications [4] and requires a troublesome work up of the acquired liver specimens. Hence, the non-invasive methods allowing reliable staging of liver scarring are now increasingly used in patients with chronic liver diseases [5–7]. Among these approaches, transient elastography (TE) [8] represents a widely used diagnostic tool, which clinical value may be improved further when used in the combination with serum surrogate markers of fibrogenesis [9, 10]. The accuracy of elastography in quantifying hepatic fibrosis has been confirmed in prospective studies [4, 5] and in meta-analyses [11], however this method is most sensitive in the setting of advanced fibrosis. On the other hand it is less accurate in staging patients with low or moderate fibrosis [12]. Measurements of serum fibrosis markers complement liver biopsy and TE. Prospective studies demonstrated that single markers (e. g., α2-macroglobulin [13], procollagen III N-peptide [9], apolipoprotein A1 [13], haptoglobin [14], hyaluronic acid [15], metalloproteinases [16]), and the AST/ALT ratio [17] allow discrimination between advanced and absent fibrosis. Since the informative value of single markers is limited, algorithms combining several markers have been introduced [18], and others proposed the combination of TE and serum markers to most accurately determine liver fibrosis [19]. However, none of the proposed marker panels has gained as much acceptance as the invasive approach [20]. This may be due to relatively high costs of marker measurements, and low sensitivity to discriminate between fibrotic, cirrhotic or steatotic liver lesions. As a result, no scores based on serum levels of hepatic fibrosis markers are actually regarded as definite methods upon which therapeutic decisions can be based.

Previous experimental data led to the identification of placental growth factor (PLGF) [21], growth differentiation factor (GDF) 15 [22] and hepatic growth factor (HGF) [23] as crucial players in hepatic fibrogenesis. For example blockade of PLGF, a specific ligand for VEGFR1 [24], ameliorates liver disease in cirrhotic mice [21]. HGF, in turn, represses the synthesis of collagen I and IV in hepatic stellate cells [23]. Since each of the above-mentioned proteins can be measured in serum, they represent possible candidate markers for liver fibrosis. Therefore in the current study, we tested for the first time panel of serum markers of liver fibrosis, including PLGF, GDF15, and HGF.

In brief, as non-invasive fibrosis tests are increasingly used in clinical practice, we aimed to evaluate the diagnostic performance of the new marker panel in a large cohort of patients with various viral and non-viral chronic liver diseases [25]. At first we compared the serum concentrations of studied markers against the classic ‘gold-standard’ method of quantifying fibrosis, namely histology (test cohort). Subsequently we transposed the results into a confirmation cohort staged non-invasively by TE.

## Study design

To determine whether circulating PLGF, GDF15, and HGF can predict hepatic fibrosis and stiffness we investigated two independent samples of patients with chronic liver diseases. Patients with available histology and available TE measurements were assigned to the test cohort. Patients with TE results only were assigned to the confirmation cohort.

In the test cohort, we determined TE cut-offs for the presence of histological liver fibrosis stage ≥F1 (F0 vs. F1/F2/F3/F4), ≥F2 (F0/F1 vs. F2/F3/F4), ≥F3 (F0/F1/F2 vs. F3/F4) and F4 (F0/F1/F2/F3 vs. F4), using ROC analysis.

Subsequently we determined the AUCs for all markers indicating significant histological liver fibrosis stages (≥F2). Cut-offs were chosen at marker concentrations with maximum sensitivity and specificity for the presence or absence of ≥F2 fibrosis (PLGF, GDF15, and HGF).

Finally we determined the distribution of patients with one, two or three markers positive (i.e. ≥ the above cut-offs) and compared the distribution between histological fibrosis stages and TE. In a second step, we evaluated the TE-staged patients only in analogy to the test cohort in order to validate the distribution of positive markers.

## Patients and methods

Overall, we prospectively recruited 834 consecutive European individuals (age 17–84 years, males n = 510) with viral (n = 559) and non-viral (n = 275) chronic liver diseases. All patients (characterised in detail in Table 1) underwent a careful clinical examination. Blood samples were drawn from fasted subjects. The study protocol followed the ethical guidelines of the declaration of Helsinki and the methods in this study were carried out in accordance to these guidelines. The local Ethics Committee of the University of Bonn and Saarland University reviewed and approved the study design and consent procedure. Written informed consent for the participation in the study was obtained from all patients.

**Table 1:** Clinical characteristics of the study cohort.

Variables	Subject characteristics	Test cohort	Validation cohort
N (males/females)	834 (510 / 324)	229 (128 / 101)	605 (381/224)
TE results	834		
TE results and liver biopsy	229		
Age (years)	51 (18–84)	54 (20–84)	49 (17–83)
BMI (kg/m^2^)	24.6 (11.3–45.9)	24.7 (11.34–45.9)	25 (15–41)
HCV vs. others (%)	499 (60%) vs. 335 (40%)	114 (49.7%) vs. 115 (50.3%)	385 (63.7%) vs. 220 (36.3%)
Specific aetiology			
HCV	499 (59.8%)	114 (49.8%)	385 (63.6%)*
Alcoholic liver disease	88 (10.6%)	55 (24.0%)	33 (5.5%)*
NASH	72 (8.6%)	6 (2.6%)	66 (10.9%)*
HBV	60 (7.1%)	9 (3.9%)	51 (8.4%)
Autoimmune hepatitis	30 (3.6%)	20 (8.7%)	10 (1.7%)*
Other liver diseases	85 (10.2%)	25 (11.0%)	60 (10.0%)*
TE (kPa)	6.8 (2.2–75.0)	20.5 (9.9–75.0)	6.5 (2.2–75.0)*
AST (U/l ±SD)	56.8 (±59.6)	63.2 (±52.4)	55.2 (±62.0)
Thrombocyte count (T/μl ±SD)	206 (±92)	183 (±112)	216 (±81)

AST: aspartate aminotransferase, BMI: body mass index, HBV: hepatitis B virus, HCV: hepatitis C virus, NASH: non-alcoholic steatohepatitis TE: transient elastography;

*P<0.05

The degree of liver fibrosis was quantified in all patients included in the study using TE (Fibroscan^®^, Echosens SA, Paris, France) as described previously [25]. From these 834 patients, a subgroup of 229 was scheduled for liver biopsy as well. This was performed using the percutaneous Menghini technique with 1.8 mm needles (Hepafix^®^ G15). Liver fibrosis was staged according to Desmet and Scheuer (i. e., scores ranging from F0 to F4) [26] by a pathologist who was blinded to TE results. Patients were only included if at least 15 portal fields were described by the pathologists.

Since to date no set of commonly accepted TE cut-offs have been established for mixed populations (i.e. including patients with viral and non-viral liver diseases), we determine the study specific TE cut-offs that correspond in our cohort to specific histologic fibrosis classes. Based on these results, all patients were stratified into five TE classes corresponding to histologic fibrosis F0, F1, F2, F3, and F4 (i.e. cirrhosis).

### Measurement of fibrosis serum markers

Serum concentrations of PLGF, GDF15 and HGF were determined in 834 patients. PLGF was tested using electrochemiluminescence test (ELECSYS PlGF, Roche Diagnostics, Mannheim, Germany). HGF was analysed using the enzyme-linked immunoassay obtained from R&D Systems (Minneapolis, USA), using a monoclonal antibody specific for HGF and a pre-coated microtiter plate. GDF15 was tested using a prototype electrochemiluminescence test containing a polyclonal GDF15 affinity chromatography-purified goat anti-human IgG antibody obtained from R&D Systems. For each of the measurement sets, a standard curve was generated with recombinant human GDF15 from R&D Systems.

### Statistical analysis

Statistical tests were performed with SPSS 20.0 (SPSS, Munich, Germany) or GraphPad Prism 5.0 (GraphPad Software Inc., CA, USA). Two-sided P-values < 0.05 were regarded as significant. Quantitative data were expressed as medians and ranges and analysed using Mann-Whitney U or Kruskal-Wallis tests, as appropriate. Qualitative traits were analysed using contingency table statistics. The correlation between serum concentrations of PLGF, GDF15 and HGF and fibrosis stages and liver stiffness were assessed by Spearman tests.

Receiver operating characteristics (ROC) analysis was applied to detect the optimal TE cut-offs and serum markers that differentiate between different histological fibrosis stages. Linear logistic regression analysis was performed to test for associations of increasing numbers of positive markers with the presence or absence of histological cirrhosis and subsequently with increased liver stiffness corresponding to cirrhosis, with P<0.05 accepting markers significantly associated with the independent variable. In addition, uni- and multivariate logistic regression analyses with stepwise forward approach were applied to define independent predictors of liver stiffness. All variables with P > 0.1 in the univariate analyses were then included in the multivariate model. Variables were included in the final multivariate model if the threshold of P < 0.05 was met.

## Results

### Determination of transient elastography cut-offs corresponding to significant fibrosis and cirrhosis

Overall, 834 patients with a median age of 51 years (range 18–84) participated in the study. Male patients were overrepresented (61.1%), and median BMI was 24.6 kg/m^2^ (range 11.3–45.9). The majority of patients suffered from chronic hepatitis C virus (HCV) infection (60.0%) (Table 1).

In a subgroup of 229 patients (defined as the test group) histological fibrosis stage and TE measurements were available. TE results correlated significantly (r = 0.739; P<0.001) with histological fibrosis stages. ROC analysis was performed in the test cohort to determine the optimal cut-off values for TE measurements (Table 2). For all further analyses, we used the TE cut-offs 9.2 kPa and 12.8 kPa, defining patients with significant liver fibrosis (i.e. ≥F2) and cirrhosis (i.e. = F4).

**Table 2:** Area under the curve and optimal cut off results for TE discriminating different fibrosis stages in the test cohort.

Histological fibrosis stage	AUC	CI	Optimal cut off for TE results	Sensitivity	Specificity
<F1 vs ≥F1	0.838	0.770–0.906*	8.7	0.62	1
<F2 vs ≥F2	0.887	0.843–0.931*	9.2	0.78	0.93
< F3 vs. ≥F3	0.916	0.876–0.955*	11.0	0.82	0.94
<F4 vs F4	0.907	0.864–0.950*	12.8	0.88	0.90

AUC: Area under the curve, CI: confidence interval, TE: transient elastography;

*P<0.05

### Serum markers discriminate histological fibrosis stages

**Fig 1:**
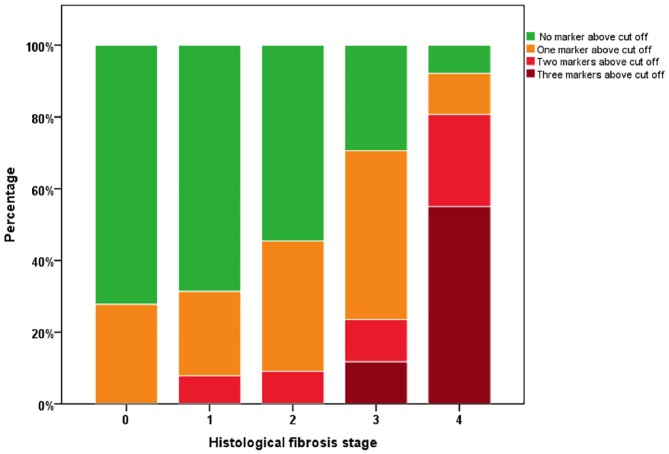
Distribution of positive markers in patients with different histological fibrosis stages. The number of positive markers correlates with advancing fibrosis.

All markers showed highly significant correlations with histological fibrosis stages (S1–S3 Figs). ROC analysis was used to define the optimal cut-off concentrations that discriminate between different histological fibrosis stages (Table 3). For further analysis, we used the serum fibrosis marker cut-offs that discriminate significant from non-significant histological fibrosis stages (i.e. <F2 vs. ≥F2), namely PLGF = 20.20 pg/ml, GDF15 = 1582.76 pg/ml and HGF = 2598.00 pg/ml. Patients with concentration of any marker equal or above the defined cut-offs were regarded "marker positive". The sensitivities and specificities to detect histological fibrosis stage ≥F2 were 79% and 63% for PLGF, 94% and 67% for GDF15, and 97% and 64% for HGF, respectively. The number of positive markers correlated significantly with histological fibrosis stages (r = 0.7; P<0.001) in the test cohort. The number of positive markers increased significantly over fibrosis stages, with the highest proportion of patients with at least one positive marker in cirrhotics (F4, 92.1%) (Fig 1). The sensitivity and specificity of at least one marker positive for presenting with at least fibrosis stage F2 was 84% and 72%, respectively. The positive predictive value of presenting with at least one marker positive and having fibrosis stage F2 was 89%. The negative predictive value was 63% with a false positive rate of 28% and a false negative rate of 16%. This indicates that patients with at least one marker positive are at high risk of developing hepatic fibrosis.

**Table 3:** Determination of AUCs and cut-offs of serum markers according to histological fibrosis stages in the test cohort.

Histological fibrosis stages	Marker	AUC	CI	Cut-off
<F1 vs ≥F1	PLGF*	0.748	0.636–0.861	18.1
	GDF15*	0.839	0.767–0.911	902.5
	HGF*	0.862	0.802–0.922	1821.3
<F2 vs ≥F2	PLGF*	0.758	0.692–0.823	20.2
	GDF15*	0.854	0.808–0.900	1582.8
	HGF*	0.849	0.802–0.898	2598.0
< F3 vs. ≥F3	PLGF*	0.771	0.710–0.832	21.9
	GDF15*	0.901	0.865–0.938	1563.7
	HGF*	0.888	0.848–0.928	2085.7
<F4 vs F4	PLGF*	0.751	0.690–0.813	23.6
	GDF15*	0.898	0.860–0.935	1822.1
	HGF*	0.899	0.861–0.938	2724.9

AUC: Area under the curve, CI: confidence interval;

*P< 0.05

### Performance of markers with respect to liver stiffness

Subsequently we evaluated the distribution of positive markers in the test cohort, with TE values ≥9.2 and ≥12.8 kPa indicating significant fibrosis and cirrhosis, respectively. Interestingly, the OR for presenting with TE ≥9.2 kPa was significantly increased for patients presenting with at least one marker above the predefined cut-off values (OR = 17.8, CI 9.0–35.2, P<0.001). This OR did not further increase in patients with TE ≥12.8 kPa, indicating that any positive marker indicates the presence of significant fibrosis but may not discriminate between significant fibrosis and cirrhosis in patients staged with TE only. However, increasing numbers of positive markers were associated with a successively increasing OR to present with TE ≥12.8 kPa, which corresponds to cirrhosis in this cohort (Table 4).

**Table 4:** Logistic regression analysis for numbers of markers corresponding to the risk of presentation with TE ≥12.8kPa in the test cohort.

Numbers of markers positive	Regression coefficient	OR	CI	P
One marker positive	1.28	3.61	1.32–9.88	<0.05
Two markers positive	3.50	33.00	10.91–99.79	<0.001
Three markers positive	4.69	108.40	30.00–391.88	<0.001

CI: confidence interval; OR: odds ratio.

Given the large numbers of patients with chronic hepatitis C virus (HCV) infection in our cohort, we evaluated HCV- and non-HCV-infected patients separately. In the HCV patients, the positivity of one, two or three markers was associated with a significantly increased OR for the presence of TE ≥9.2 kPa (one marker: OR = 4.7, CI 2.8–8.0, P<0.001; two markers: OR = 9.9, CI 5.4–18.2, P<0.001; three markers: OR = 59.9, CI 23.4–153.4, P<0.001). This association was similarly present n patients without the HCV infection (one marker: OR = 3.9, CI 1.9–8.2, P<0.001; two markers: OR = 18.3, CI 7.8–42.7, P<0.001; three markers: OR = 144, CI 59–3383, P<0.001). The markers also discriminated patients with TE ≥12.8 kPa from patients with TE <12.8 kPa in HCV-infected patients (one marker: OR = 6.6, CI 3.0–14.5, P<0.001; two markers: OR = 19.8, CI 8.8–44.5, P<0.001; three markers: OR = 130, CI 47–356, P<0.001) and patients without HCV infection (one marker: OR = 5.4, CI 2.1–14.5, P<0.001; two markers: OR = 20.3, CI 7.8–52.6, P<0.001; three markers: OR = 155, CI 46–520, P<0.001).

To address the question whether the presence of positive markers adds information in patients with TE values (i.e. <9.2 kPa), we used contingency tables. Among 93 patients with low TE values, 30 (33%) presented with fibrosis stages ≥F2 and would have been missed by TE alone. The presence of any marker positive allowed however the detection of 15 (50%) of these cases. False positive were only 14 out of 94 patients (15%). Thus, any marker positive can be considered as stand-alone risk factor for the presence of significant fibrosis.

### Evaluation of serum markers in the confirmation cohort

**Fig 2:**
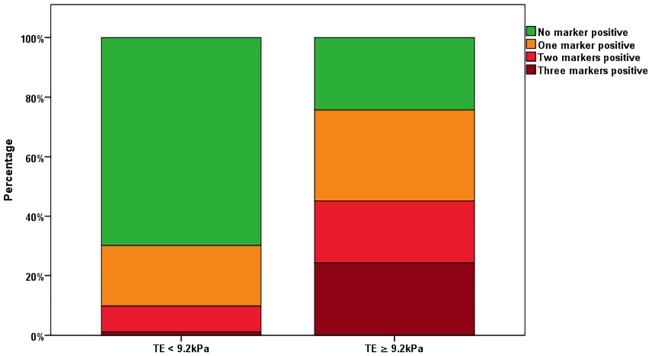
Numbers of positive markers corresponding to patients with Transient Elastography (TE) measurements equal or above 9.2 kPa or below 9.2 kPa.

The number of positive markers correlated (r = 0.49; P<0.001) with TE measurements also in the confirmation cohort. Interestingly, patients presenting with TE ≥9.2kPa showed a marked increase in numbers of positive markers (Fig 2). The OR for presenting with TE ≥9.2 kPa was significantly increased for patients with at least one marker positive (OR = 7.3, CI 4.7–11.3, P<0.001). Similarly to our observation in the test cohort, we found an increase of the OR for the presence of TE≥12.8 kPa with increasing numbers of positive markers (Table 5).

**Table 5:** Logistic regression analysis for numbers of markers corresponding to the risk of presentation with TE ≥12.8kPa in the validation cohort.

Numbers of markers positive	Regression coefficient	OR	CI	P
One marker positive	1.91	6.75	3.29–13.83	<0.001
Two markers positive	2.51	12.29	5.68–26.60	<0.001
Three markers positive	4.70	111.00	41.89–288.84	<0.001

CI: confidence interval; OR: odds ratio.

In order to define independent predictors of significant fibrosis (corresponding to TE ≥9.2 kPa), we performed uni- and multivariate regression analyses. Variables that entered the uni-variate analysis were age, gender, BMI, and the number of positive markers. Whereas in the uni-variate model classical co-factors of fibrosis proved to be significantly associated with TE (Table 6), only the number of markers and BMI were independently associated with the risk of significant liver stiffness in the multi-variate analysis (Table 7).

**Table 6:** Uni-variate logistic regression analysis for the presence of increased liver stiffness (≥ 9.2kPa) corresponding to significant fibrosis (≥ F2) in the validation cohort.

Variable	Regression coefficient	OR	CI	P
Age	0.039	1.040	1.028–1.052	<0.001
Gender (Reference female)	0.312	1.366	1.018–1.833	0.038
BMI	0.064	1.066	1.025–1.108	0.001
Numbers of markers positive (Reference: no marker positive)				
One marker positive	1.488	4.429	2.905–6.752	<0.001
Two markers positive	2.469	11.810	7.294–19.124	<0.001
Three markers positive	4.769	117.760	51.930–267.039	<0.001

BMI: body mass index; CI: confidence interval; OR: odds ratio.

**Table 7:** Multivariate logistic regression analysis for the presence of increased liver stiffness (≥ 9.2kPa) corresponding to significant fibrosis (≥ F2) in the validation cohort.

Variable	Regression coefficient	OR	CI	P
Age	0.013	1.013	0.995–1.031	0.160
Gender (Reference female)	-0.365	0.694	0.442–1.091	0.113
BMI	0.075	1.078	1.025–1.133	0.004
Numbers of markers positive (Reference: no marker positive)				
One marker positive	1.397	4.043	2.463–6.637	<0.001
Two markers positive	2.336	10.338	5.860–45.981	<0.001
Three markers positive	4.744	114.917	45.981–287.205	<0.001

BMI: body mass index; CI: confidence interval; OR: odds ratio.

## Discussion

In this study, including over 800 patients with chronic liver diseases, we availed of a novel set of markers that may be used to further define patients with chronic liver diseases at-risk of advanced fibrosis. As shown in Table 1, the majority of individuals included in our cohort suffered from chronic HCV infection, however we were able to recruit patients with a large variety of other liver diseases. Hence, the three-marker algorithm that we established might be useful in the setting of a wide range of chronic liver diseases and assist in the management of antiviral treatment as well as the initiation of antifibrotic therapies.

Our study is the first to implement all three markers for clinical diagnosis of significant fibrosis and cirrhosis. Interestingly, a recent report of an association of GDF15 in an Asian population confirmed the association with cirrhosis despite a slightly higher cut-off [27]. The panel of serum markers defined in our study adds further non-invasive options to characterize patients at risk for liver fibrosis. Of note, the serum concentrations of PLGF, HGF and GDF15 correlated with hepatic fibrosis assessed both non-invasively by TE and semi-quantitatively by liver biopsy. Moreover, the multivariate regression analyses detected increased marker concentrations as determinants of liver stiffness that were independent from other potentially profibrogenic factors. ORs for presenting with advanced fibrosis and/or liver stiffness values that correspond to cirrhosis range as high as 111 when three markers are positive. This underlines the potential of these markers to improve other non-invasive fibrosis tests, either ultrasound- or serum marker-based.

As demonstrated in the supplementary material, not all three markers showed a steady increase of their serum concentrations with increasing fibrosis stages. This observation might reflect the notion that fibrogenesis is a complex process involving both destructive and remodelling mechanisms. Therefore markers with distinct effects on regeneration, fibrosis progression and regression may be involved in early or late stages of liver fibrosis. Of note, all our patients had ongoing liver disease, and the dynamics of the three markers might demonstrate their different effects on fibrogenesis.

We demonstrate that measurements of serum markers in patients with chronic liver diseases add further info to the results obtained non-invasively by TE. Especially patients with false negative TE results, regardless of high degree of liver scarring, might be identified with the panel of three markers we tested in the current study.

In addition, we show that the marker set might be useful for monitoring hepatic fibrogenesis, since we detected a stepwise increase of positive markers with increasing fibrosis stages and liver stiffness. Although selected markers demonstrated slightly higher AUCs for the presence of specific histological fibrosis stages, we were unable to identify a single marker that was more accurate than any other marker of the set. As with TE measurements, each single marker shows a large range of possible values when plotted against the semiquantitative fibrosis stages (data not shown). Therefore, the discrimination of definite fibrosis stages solely based on serum marker levels is not feasible. However, as has been shown for other scores (e.g. model of end stage liver disease), it is helpful to combine markers in a set to further improve the diagnostic performance of a test.

To our knowledge this is the first translational study that demonstrates the clinical relevance of these so far experimental fibrosis markers, indicating that the role of the three markers in the pathobiology of fibrogenesis should be investigated further. PLGF is a cytokine that is over-expressed in cirrhotic liver tissue, and van Steenkiste et al. [21] demonstrated that PLGF serum concentrations are increased in patients with liver cirrhosis, whereas *Plgf*^-/-^ mice display decreased portal pressure after induction of severe fibrosis with CCl_4_ [21]. HGF inhibits the accumulation of extracellular matrix proteins in liver and modulates the progression of fibrosis [23, 28, 29]. With respect to GDF15, a study by Hsiao et al. [22] demonstrated that its expression is substantially induced after liver injury and during hepatic regeneration. Taken together each of these markers represents a modulator of hepatic fibrosis, however our analysis is the first to underline their combined clinical value as significant predictors of hepatic fibrosis in humans.

Liver fibrogenesis and regression of liver fibrosis is characterised by profibrogenic mechanisms such as TGF-beta overexpression and anti-fibrotic mechanisms such as metalloproteinase expression [30]. While overexpression of HGF may have beneficial effects in fibrosis models and GDF15 levels potentially reflect healing capacities [27]. PLGF seems to be a profibrogenic factor that can be targeted by antifibrotic therapies [21]. It is potentially wise to address all of these aspects of fibrosis in future non-invasive monitoring during antifibrotic therapies. The three markers presented here share these characteristics and should therefore be evaluated in both population-based observational studies as well as antifibrotic treatment trials.

Our study has some limitations. First of all, liver biopsy remains the gold-standard-method of measuring liver fibrosis. Since liver biopsy results were not available in all patients included in the study, we had to rely on liver stiffness as the non-invasive tool to quantify liver fibrosis in the confirmation cohort. Secondly, even if the studied group of patients was relatively large, we did not have enough power to investigate if either of the markers has disease-specific cut-offs to detect different grades of liver fibrosis. Liver stiffness measurements are prone to false increase due to cholestasis, flares and non-fasting condition. Hence, we excluded patients with these conditions. As the result, we have no information whether our marker set is reliable under these conditions and conclusions based on our results are limited to patients with stable liver diseases. Finally, in our study we focussed on the assessment of three relatively new fibrosis serum markers predominantly in relation to a TE-based quantification of liver injury. Using cut-offs and categorising patients according to these cut-offs may lead to a loss of information, even if this strategy might be more applicable in clinical practice then using continuous variables. We recon that future analysis of PLGH, GDF15 and HGF in relation to tools allowing quantification of liver injury as continuous traits might allow even more complex assessments using sophisticated models (e.g. LASSO analysis)

In conclusion, in this study we describe the potential benefits of three serum markers for the detection of patients with advanced fibrosis in a cohort of patients with liver biopsy and a validation cohort. The risks described by the combinations of these markers were independent from other classical fibrosis risk factors. Moreover, the panel adds information to TE, especially in patients with low TE values. Last but not least, the set of markers may be a useful tool to monitor patients with chronic liver diseases during and after therapy.

## Supporting information

S1 Fig Distribution of the GDF15 values according to histological fibrosis stages. (TIF)

S2 Fig Distribution of the HGF values according to histological fibrosis stages. (TIF)

S3 Fig Distribution of the PLGF values according to histological fibrosis stages. (TIF)
