# Correction: Panel of three novel serum markers predicts liver stiffness and fibrosis stages in patients with chronic liver disease

**DOI:** 10.1371/journal.pone.0179205

**Published:** 2017-06-02

**Authors:** Marcin Krawczyk, Simone Zimmermann, Georg Hess, Robert Holz, Marc Dauer, Jochen Raedle, Frank Lammert, Frank Grünhage

The following information is missing from the Funding Section: FL received funding from the Federal Ministry of Education and Research (BMBF LiSyM 031L0051).

There is an error in the first sentence of the Patients and methods section. The correct sentence is: Overall, we prospectively recruited 834 consecutive European individuals (age 18–84 years, males n = 510) with viral (n = 559) and non-viral (n = 275) chronic liver diseases.

In the Patients and methods section, there is an error in the penultimate sentence under the subheading “Statistical analysis.” The correct sentence is: All variables with P < 0.1 in the univariate analyses were then included in the multivariate model.

In the Results section, there is an error in the ninth sentence under the subheading “Serum markers discriminate histological fibrosis stages.” The correct sentence is: The positive predictive value of presenting with at least one marker positive and having fibrosis stage ≥ F2 was 89%.

In the Results section, there is an error in the seventh sentence under the subheading “Performance of markers with respect to liver stiffness.” The correct sentence is: This association was similarly present in patients without HCV infection (one marker: OR = 3.9, CI 1.9–8.2, P<0.001; two markers: OR = 18.3, CI 7.8–42.7, P<0.001; three markers: OR = 144, CI 59–3383, P<0.001).

In the Results section, there is an error in the ninth sentence under the subheading “Performance of markers with respect to liver stiffness.” The correct sentence is: To address the question whether the presence of positive markers adds information in patients with low TE values (i.e. <9.2 kPa), we used contingency tables.
