# Supplementary figures and images for: Panel of three novel serum markers predicts liver stiffness and fibrosis stages in patients with chronic liver disease
Source: PLoS One. 2017 Mar 16;12(3):e0173506. doi: 10.1371/journal.pone.0173506 (PMC5354278; doi:10.1371/journal.pone.0173506)

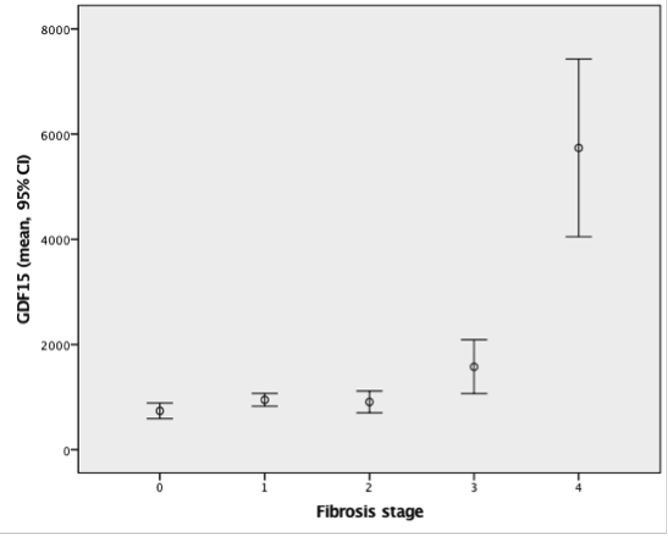

Supplement: S1 Fig — (TIF) [file pone.0173506.s001.tif]

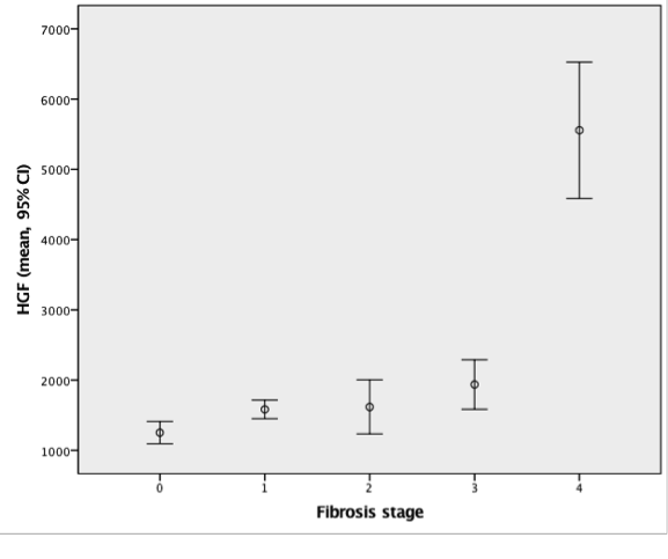

Supplement: S2 Fig — (TIF) [file pone.0173506.s002.tif]

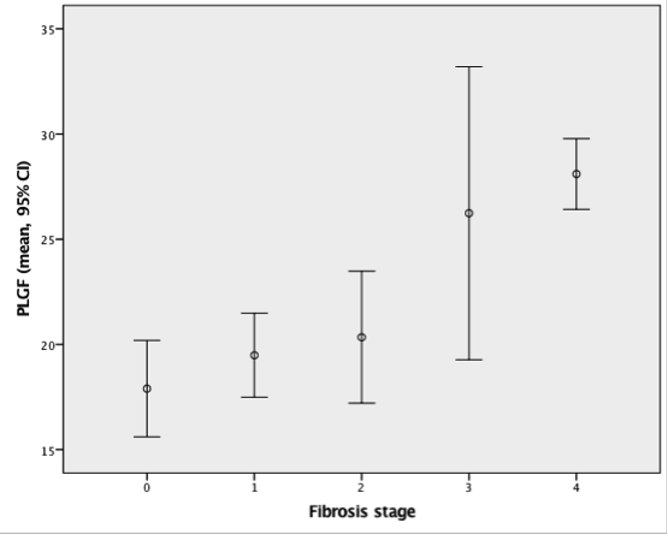

Supplement: S3 Fig — (TIF) [file pone.0173506.s003.tif]
